# Association between brachial-ankle pulse wave velocity and the risk of new-onset atrial fibrillation: A report from Kailuan prospective cohort study

**DOI:** 10.1038/s44325-023-00001-7

**Published:** 2024-03-06

**Authors:** Wenhua Song, Zongshuang Song, Nan Zhang, Meijuan Zhang, Gary Tse, Oscar Hou In Chou, Guangping Li, Gan-Xin Yan, Gregory Y. H. Lip, Shouling Wu, Tong Liu

**Affiliations:** 1 Tianjin Key Laboratory of Ionic-Molecular Function of Cardiovascular Disease, Department of Cardiology, Tianjin Institute of Cardiology, Second Hospital of Tianjin Medical University, 300211 Tianjin, China; 2 School of Public Health, North China University of Science and Technology, Tangshan, China; 3 Epidemiology Research Unit, Cardiovascular Analytics Group, Hong Kong, China–UK Collaboration, Hong Kong, China; 4 Kent and Medway Medical School, Canterbury, Kent, CT2 7NZ UK; 5 School of Nursing and Health Studies, Hong Kong Metropolitan University, Hong Kong, China; 6 Division of Clinical Pharmacology and Therapeutics, Department of Medicine, School of Clinical Medicine, Li Ka Shing Faculty of Medicine, University of Hong Kong, Hong Kong, China; 7 Lankenau Medical Center and Lankenau Institute for Medical Research, Wynnewood, PA USA; 8 Fuwai Huazhong Hospital, Zhengzhou, Henan China; 9 Sidney Kimmel Medical College at Thomas Jefferson University, Philadelphia, PA USA; 10 Liverpool Centre for Cardiovascular Sciences, University of Liverpool, Liverpool John Moores University and Liverpool Heart & Chest Hospital, Liverpool, UK; 11 Department of Clinical Medicine, Aalborg University, Aalborg, Denmark; 12 Department of Cardiology, Kailuan General Hospital, Tangshan, China

**Keywords:** Cardiology, Diseases

## Abstract

One marker of arterial stiffness (AS) is the brachial-ankle pulse wave velocity (baPWV). We aim to investigate the predictive value of baPWV with regard to new-onset atrial fibrillation (AF). All participants without AF from 2010 to 2020 in the Kailuan cohort were included. The primary endpoint was new-onset AF. Participants were categorized into three study groups based on baPWV, with a normal baPWV group as a reference. The predictive value of baPWV was analyzed as a continuous variable. Multivariable Cox proportional hazard regression models were used to investigate the association. A total of 49,872 subjects (mean age: 47.57 years old, 74.2% male) were included with a mean follow-up of 6.17 (3.95–8.46) years. The risk of AF increased as the baseline baPWV increased, whereby the adjusted hazard ratio (aHR) of the borderline AS group and the elevated AS group were 1.82 (95% confidence interval [CI]: 1.18–2.80) and 2.08 (95% CI: 1.31–3.30), respectively. When considered as a continuous variable, each 361 cm/s increase in baseline baPWV, increased the risk of AF by 21.7% (aHR: 1.22; 95% CI: 1.08–1.37). In the subgroup analysis of non-hypertensive patients, the risks of AF were significantly higher in the borderline AS group (aHR: 3.16, 95% CI: 1.74–5.74) and elevated AS group (aHR: 2.26, 95% CI: 1.02–5.05). For patients with elevated BMI, the risk of AF in the elevated AS group was significantly higher (aHR: 1.69, 95% CI: 1.00–2.83). Baseline baPWV was associated with new-onset AF after adjustments. (Trial registration site and registration number are, respectively, http://www.chictr.org.cn/index.aspx and ChiCTR-TNRC-11001489).

## Introduction

Atrial fibrillation (AF) is the most common sustained cardiac arrhythmia observed in clinical practice. Its prevalence increases with age and is associated with an increased risk of mortality and morbidity, as well as hospitalizations and healthcare expenditures^1^. Arterial stiffness (AS) is one of the key risk factors for cardiovascular disease and mortality in high-risk cardiovascular patients^2^. There are many shared risk factors between AF and AS, such as age, male, hypertension, valvular heart disease, left ventricular insufficiency, obesity, alcohol, smoking, diabetes, and obstructive sleep apnea; for instance, endothelial dysfunction is described as the common underlying pathophysiological feature^3,4^.

Several non-invasive methods have been developed to evaluate AS. One such method is the brachial-ankle pulse wave velocity (baPWV), which is a clinical index reflecting vascular stiffness. The baPWV has been used as a predictor of cardiovascular outcomes in the general population, as well as in patients with hypertension, diabetes, or end-stage renal disease^5^. Unlike the carotid-femoral pulse wave velocity (cfPWV) which measures the stiffness of the central arteries, baPWV is a comprehensive measurement of the stiffness of both the central and peripheral arteries. BaPWV is easy to measure clinically and requires only the use of cuffs on the limbs without exposing the groin area, making it a popular method for non-invasive assessment of AS. However, while previous studies mainly focused on cardiac outcomes related to vascular diseases, information on the relationship between baPWV and new-onset AF is lacking.

The aim of this study was to investigate the predictive value of baPWV for new-onset AF.

## Methods

### Study participants

The study was conducted in accordance with the Helsinki Declaration and approved by the Ethics Committee of Kailuan General Hospital (Registration number: ChiCTR-TNRC-11001489); written informed consents were obtained from all participants. This was a prospective observational cohort study using the Kailuan cohort, which has been extensively described previously^6,7^. This cohort enrolled participants from 11 hospitals responsible for community healthcare. The inclusion criteria were subjects who participated in baPWV examinations, were clear of AF at baseline, and provided written informed consent. The exclusion criteria were as follows: (i) patients with a history of AF or cryptogenic stroke at baseline; (ii) patients without regular follow-up and physical examination data in the same period (missing physical examination data within four years).

## Data collection

The registration methods of demographic data, including smoking, alcohol status, salt intake, concomitant diseases and blood markers, have been detailed previously^6,8^. Self-report proforma was used to determine smoking and alcohol status (yes vs no). Body mass index (BMI) is defined as weight (kg)/height (m)^2^. According to BMI, patients were divided into non-elevated BMI group (BMI < 24.0 kg/m²); and elevated BMI group (BMI ≥ 24.0 kg/m^2^). Sitting systolic blood pressure (SBP) and diastolic blood pressure (DBP) were measured with either a mercury sphygmomanometer or electronic sphygmomanometer, and the average of three consecutive readings was calculated. Hypertension was defined as blood pressure ≥ 140/90 mmHg, self-reported history of hypertension, or the use of any antihypertensive medication. The laboratory testing results were also collected, including blood lipid, high-sensitivity C-reactive protein (hs-CRP), and so forth; the specific determination methods have been detailed in the previous literature. All plasma samples were measured with the autoanalyzer (Hitachi 747)^6,8^.

### BaPWV measurement

BaPWV was measured by a BP-203 RPE III networked arterial stiffness detection device (Omron Health Medical [China] Co., Ltd.). The room temperature was kept at 22–25 °C during the examination. Participants were instructed to refrain from smoking, drinking alcohol, coffee, or engaging in intense physical activity for 24 h and were allowed 15 min to rest before the examination. During the examination, the observed object remained supine, the upper arm cuff airbag mark was aligned with the brachial artery, and the lower limb cuff airbag mark was located on the inside of the lower limb. Each subject was measured twice, and the second data recording was taken as the final result. In this study, the larger value of baPWV on the left or the right sides was analyzed as detailed elsewhere^6,8^. We divided the participants into three groups according to baPWV: (i) normal arterial stiffness: baPWV < 1400 cm/s; (ii) borderline arterial stiffness: 1400 cm/s ≤ baPWV < 1800 cm/s; and (iii) elevated arterial stiffness: baPWV ≥ 1800 cm/s^6^.

### Follow‑up and assessment of AF

All participants without AF from 2010 to 2020 in the Kailuan cohort were included and were followed up until December 31, 2020, or until they were diagnosed with AF or passed away. Cardiovascular events in the Kailuan study were determined by reviewing the local hospitals’ annual discharge list, the death certificate of the State Office, and annual participant surveys about the history of cardiovascular events. The primary endpoint was new-onset AF during follow-up. In the biennial office visit, participants were investigated with the 12-lead electrocardiogram (ECG) in the supine position after resting in a quiet room for at least 5 min. ECGs interpretations were confirmed by two cardiologists, as previously described^9^. The electrocardiographic diagnostic criteria for AF included: (1) irregular RR intervals; (2) absence of P-waves; and (3) irregular atrial activity, according to the European Society of Cardiology guidelines^10^.

### Statistical methods

The data were analyzed using SAS 9.4 (SAS Institute, Cary, NC, USA). Continuous variables were presented as mean ± standard deviation, and the analysis of variance was used for comparison between groups; median (interquartile range) was used for variables with a skewed distribution, and the Kruskal–Wallis test was used to analyze group differences. Categorical variables were presented as *n* (%), and the *χ*^2^ test was used for comparisons between groups.

Multivariable Cox proportional hazard regression models were constructed to estimate the association of baPWV with the risk of AF. Model 1 was adjusted for age and sex at baseline. Model 2 was further adjusted for education level, smoking, alcohol status, physical activity, salt intake, BMI, mean arterial pressure (MAP), fasting blood glucose (FBG), low-density lipoprotein cholesterol (LDL-C), high-density lipoprotein cholesterol (HDL-C), and total cholesterol (TC) at baseline. Model 3 was further adjusted for the use of antihypertensive, antidiabetic, and lipid-lowering medications.

To explore the relationship between baPWV and AF, subgroup analyses were performed according to age, gender, hypertension, and BMI. In each subgroup, the normal group was used as the reference. Sensitivity analysis was conducted by excluding the participants whose components of the baPWV group changed during the follow-up period. The Kaplan–Meier method was performed to evaluate the incidence rate of AF, and differences between groups were evaluated using the log-rank test. A two-sided *P* < 0.05 was considered statistically significant. We also used restricted cubic splines to evaluate the potential linear relationship between baPWV and AF.

## Results

### Baseline characteristics

**Fig. 1:**
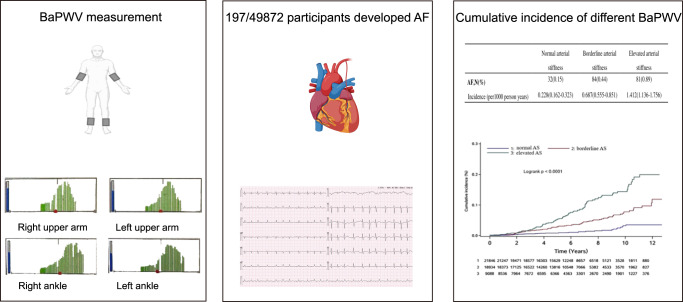
Brachial-ankle pulse wave velocity and the risk of new-onset atrial fibrillation. A total of 49,872 subjects were followed up for 6.17 (3.95–8.46) years and 197 participants developed AF eventually. Compared with the reference group, the risk of AF increased with increasing baseline baPWV. AF atrial fibrillation, BaPWV brachial-ankle pulse wave velocity, AS arterial stiffness.

**Fig. 2:**
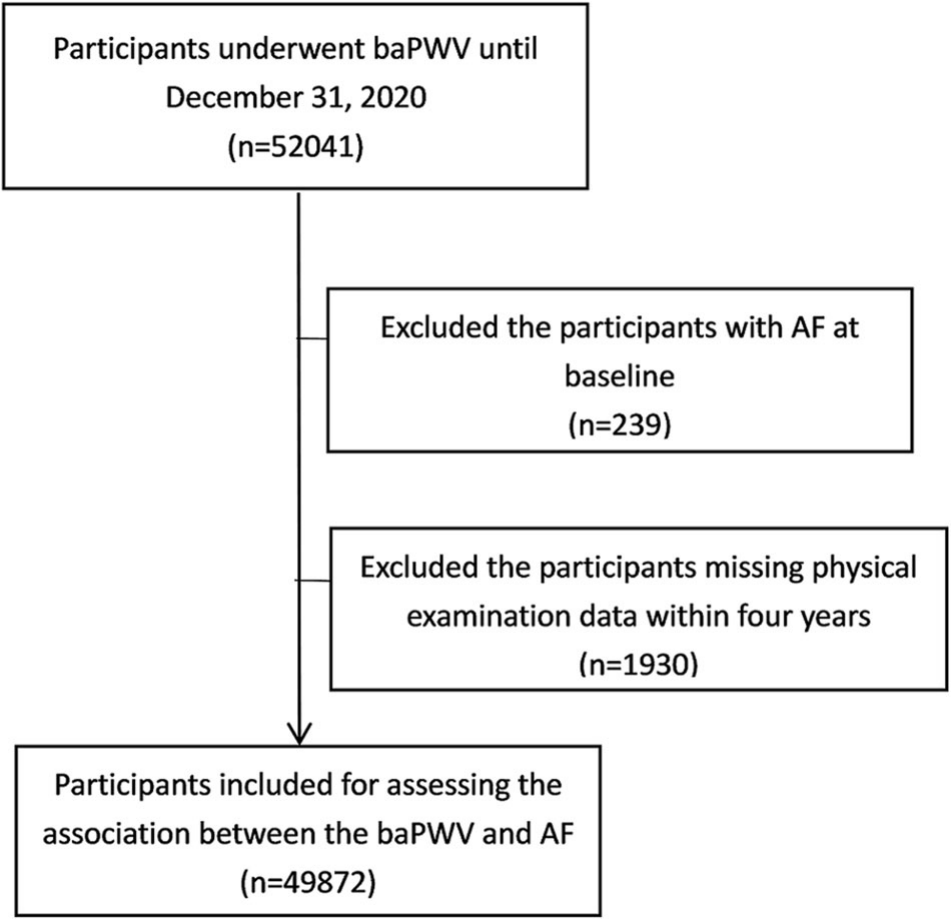
Flow chart showing the patient selection process. BaPWV brachial-ankle pulse wave velocity, AF atrial fibrillation.

A total of 49,872 subjects were enrolled in this study for a mean followed of 6.17 (3.95–8.46) years from 2010 to 2020 (Fig. 1), after excluding 239 patients with AF at baseline and 1930 patients with missing examination data (Fig. 2). The median age was 47.57 years, and 37,021 (74.2%) were male. The baseline characteristics of patients with different levels of baPWV are shown in Table 1. 197 participants eventually developed AF (0.4%). Compared to participants with a normal baPWV, those with a higher baPWV were older, with more males, less educated, and had a larger percentage of smoking, alcohol intake, salt intake, and lack of physical activity. Furthermore, regarding other clinical features, the higher baPWV group had higher BMI levels, a lower eGFR, and more coronary heart disease, diabetes, hypertension, and dyslipidemia. Additionally, this group had higher levels of SBP, DBP, FBG, TC, TG, LDL-C, and hs-CRP, but a lower level of HDL-C.

**Table 1:** The participant’s characteristics in the different levels of baPWV (*N* = 49,872)

Covariates	Total (*N* = 49,872)	Normal arterial stiffness (*N* = 21,846)	Borderline arterial stiffness (*N* = 18,935)	Elevated arterial stiffness (*N* = 9091)	*P* value
Age, years	47.57 ± 14.37	40.16 ± 11.39	49.70 ± 12.92	60.94 ± 12.35	<0.01
Men	37,021 (74.2)	13,777 (63.1)	15,718 (83.0)	7526 (82.8)	<0.01
High school education or above	19,342 (38.8)	11,206 (51.3)	5997 (31.7)	2139 (23.5)	<0.01
*Smoking status*					<0.01
No	40,024 (80.3)	18,361 (84.0)	14,552 (76.9)	7111 (78.2)	
Yes	9848 (19.7)	3485 (16.0)	4383 (23.1)	1980 (21.8)	
*Drinking status*					<0.01
No	29,658 (59.5)	14,181 (64.9)	10,349 (54.7)	5128 (56.4)	
Yes	20,214 (40.5)	7665 (35.1)	8586 (45.3)	3963 (43.6)	
*Salt intake*					<0.01
≤10, g/day	45126 (90.5)	20,021 (91.6)	16,994 (89.7)	8111 (89.2)	
>10, g/day	4746 (9.52)	1825 (8.35)	1941 (10.3)	980 (10.8)	
Physical activity, *N*(%)	37,563 (75.3)	16,057 (73.5)	14,308 (75.6)	7198 (79.2)	<0.01
BMI, kg/m^2^	24.99 ± 3.49	24.41 ± 3.58	25.44 ± 3.35	25.43 ± 3.34	<0.01
eGFR, ml/min/1.73 m^2^	98.92 ± 22.90	103.93 ± 23.37	97.55 ± 22.01	89.76 ± 20.19	<0.01
Hs-CRP, mg/L	1.30 (0.64–2.90)	1.10 (0.52–2.42)	1.40 (0.70–3.00)	1.70 (0.84–3.60)	<0.01
TG, mmol/L	1.29 (0.87–2.02)	1.11 (0.77–1.72)	1.42 (0.96–2.22)	1.45 (1.02–2.24)	<0.01
TC, mmol/L	4.92 (4.30–5.60)	4.71 (4.14–5.35)	5.06 (4.43–5.73)	5.17 (4.50–5.90)	<0.01
LDL-c, mmol/L	2.71 (2.20–3.26)	2.59 (2.11–3.10)	2.80 (2.28–3.35)	2.87 (2.28–3.46)	<0.01
HDL-c, mmol/L	1.37 (1.16–1.63)	1.39 (1.14–1.62)	1.35 (1.14–1.60)	1.36 (1.14–1.62)	<0.01
SBP, mmHg	130.83 ± 19.06	120.79 ± 14.66	134.50 ± 16.41	147.29 ± 19.39	<0.01
DBP, mmHg	81.80 ± 11.00	77.38 ± 9.54	84.42 ± 10.34	86.94 ± 11.60	<0.01
FBG, mmol/L	5.30 (4.87–5.95)	5.09 (4.71–5.51)	5.41 (4.97–6.11)	5.90 (5.20–7.56)	<0.01
ASCVD	1726 (3.46)	244 (1.12)	697 (3.68)	785 (8.63)	<0.01
Hypertension	18,969 (38.0)	3297 (15.1)	8919 (47.1)	6753 (74.3)	<0.01
Diabetic	7167 (14.4)	1020 (4.67)	3071 (16.2)	3076 (33.8)	<0.01
Hyperlipemia	17,849 (35.8)	5911 (27.1)	7709 (40.7)	4229 (46.5)	<0.01
Antihypertensive agent use	10,743 (21.5)	3134 (14.3)	4222 (22.3)	3387 (37.3)	<0.01
Antidiabetic medication use	3126 (6.27)	345 (1.58)	1285 (6.79)	1496 (16.5)	<0.01
Lipid-lowering medication use	3150 (6.32)	452 (2.07)	1343 (7.09)	1355 (14.9)	<0.01

*BMI* body mass index, *TC* total cholesterol, *TG* Triglycerides, *LDL-c* low-density lipoprotein cholesterol, *HDL-c* high-density lipoprotein cholesterol, *SBP* systolic blood pressure, *DBP* diastolic blood pressure, *FBG* fasting blood glucose, *eGFR* estimated glomerular filtration rate, *hs-CRP* high-sensitivity C-reactive protein, *ASCVD* atherosclerotic cardiovascular diseases.

### Association of baPWV with AF

**Fig. 3:**
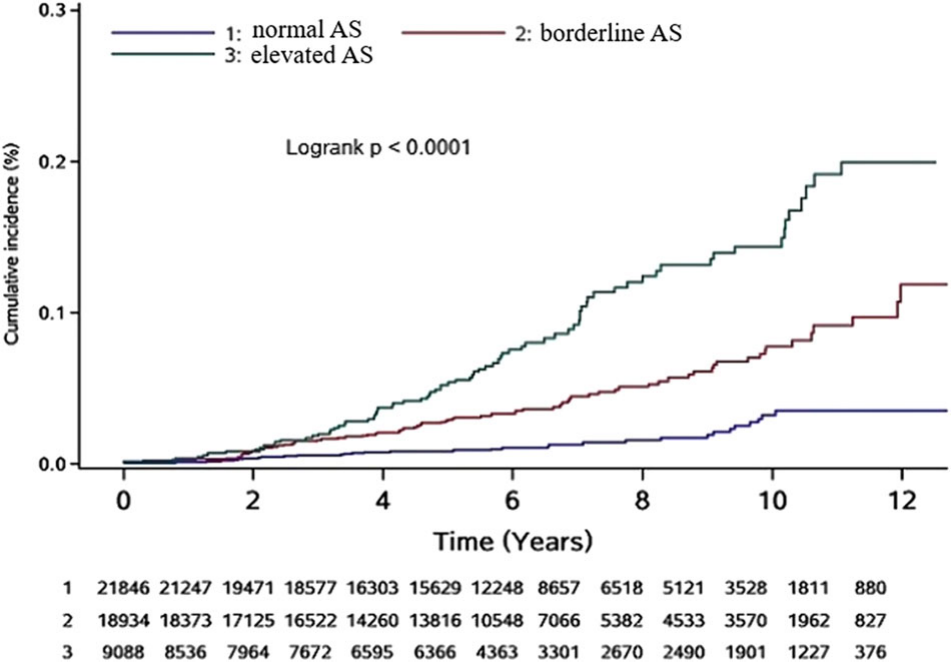
Cumulative incidence of different baPWV groups. Log-rank test was used to test for any difference across the groups. AS, arterial stiffness.

During follow-up, 197 participants (0.4%) developed AF. The relationship between baPWV and the risk of AF is shown in Table 2. The incidence of AF increased significantly with baPWV, from 0.23 (95% CI: 0.16–0.32) per 1000 person-years in the reference group to 1.41 (95% CI: 1.14–1.76) per 1000 person-years in the elevated AS group. The Kaplan–Meier curves showed that participants in the elevated AS group had a higher risk of AF than the other two groups during the follow-up period (log-rank test: *P* < 0.0001; Fig. 3).

**Table 2:** Adjust hazard ratios for incident AF in relation to different levels of baPWV

	Normal arterial stiffness	Borderline arterial stiffness	Elevated arterial stiffness	Increase 1 SD
AF, *N*(%)	32 (0.15)	84 (0.44)	81 (0.89)	
Incidence (per1000 person-years)	0.228 (0.162–0.323)	0.687 (0.555–0.851)	1.412 (1.136–1.756)	
Model 1	ref	2.268 (1.498–3.434)	2.846 (1.879–4.310)	
Model 2	ref	1.938 (1.261–2.979)	2.282 (1.449–3.593)	
Model 3	ref	1.816 (1.180–2.796)	2.079 (1.311–3.297)	1.217 (1.079–1.373)

Model 1: adjusted for age and gender.

Model 2: adjusted for age, gender, education, smoking, alcohol status, physical activity, salt intake, BMI, mean arterial pressure (MAP), fasting blood glucose (FBG), low-density lipoprotein cholesterol (LDL-c), high-density lipoprotein cholesterol (HDL-c) and total cholesterol (TC).

Model 3: adjusted for all the variables in model 2 and antidiabetic treatment, antihypertensive treatment, and lipid-lowering treatment.

*BaPWV* brachial-ankle pulse wave velocity, *AF* atrial fibrillation, *SD* standard deviation.

The risk of AF increased with the baseline baPWV, and the results remained significant after adjusting for age, sex, education level, smoking, alcohol status, physical activity, salt intake, BMI, FBG, LDL-C, HDL-C and TC, and use of antihypertensive, antidiabetic and lipid-lowering medications in model 3. Compared to the reference group, the fully adjusted HR of the borderline AS group and the elevated AS group (model 3) were 1.82 (95% CI: 1.18–2.80) and 2.08 (95% CI: 1.31–3.30), respectively. The result suggested that the adjusted risk of AF in the elevated AS group was almost twice as high as in the reference group (Table 2).

**Fig. 4:**
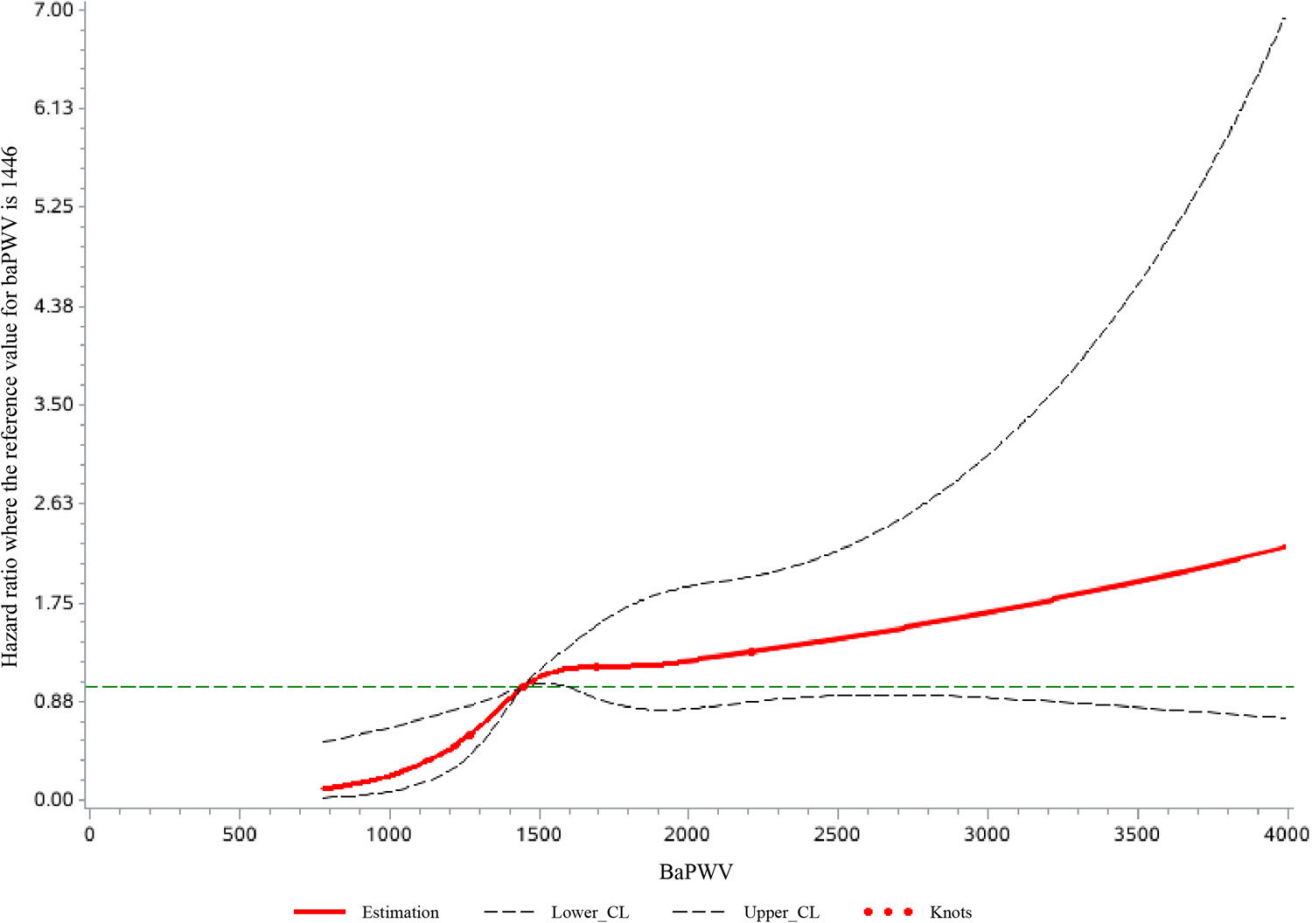
Association between baPWV and risk of atrial fibrillation. The association was adjusted for age, sex, education, smoking, alcohol status, physical activity, salt intake, BMI, MAP, FBG, LDL-c, HDL-c, TC, antidiabetic drugs, lipid-lowering drugs and antihypertensive drugs. The baPWV of 1446 cm/s was taken as the reference value, at which point HR was 1. The plot showed a lower level of risk within the lower range of baPWV and then increased thereafter. BaPWV brachial-ankle pulse wave velocity.

In the sensitivity analysis, the result remained consistent after excluding participants with changes in baPWV grouping during the follow-up period (*n* = 6886) (Table 3). Besides, when baPWV was treated as a continuous variable, for every 1 standard deviation increase in baseline baPWV, which is equivalent to 361 cm/s, the risk of AF increased by 21.7% (HR: 1.22, 95% CI: 1.08–1.38) (Table 2). Furthermore, the multivariable linear regression model showed a J-shaped association between baPWV and the risk of AF. Specifically, in the lower range of baPWV, the risk of AF was low with a steep slope. However, when baPWV exceeded 1446 cm/s, the AF risk still increased with baPWV but with a relatively flattened slope (Fig. 4).

**Table 3:** Sensitive analysis for hazard ratios values and 95% confidence intervals according to levels of baPWV^a^

	Normal arterial stiffness	Borderline arterial stiffness	Elevated arterial stiffness
AF, *N*(%)	25 (0.13)	68 (0.43)	72 (0.89)
Incidence rate (per1000 person-years)	0.211 (0.142–0.312)	0.693 (0.546–0.879)	1.447 (1.148–1.823)
Model 1	ref	2.422 (1.517–3.868)	3.064 (1.932-4.858)
Model 2	ref	2.088 (1.236–3.263)	2.387 (1.440–3.958)
Model 3	ref	1.935 (1.189–3.147)	2.269 (1.358–3.791)

Model 1: adjusted for age and gender.

Model 2: adjusted for age, gender, education, smoking, alcohol status, physical activity, salt intake, BMI, mean arterial pressure (MAP), fasting blood glucose (FBG), low-density lipoprotein cholesterol (LDL-c), high-density lipoprotein cholesterol (HDL-c) and total cholesterol (TC).

Model 3: adjusted for all the variables in model 2 and antidiabetic treatment, antihypertensive treatment and lipid-lowering treatment.

*BaPWV* brachial-ankle pulse wave velocity, *AF* atrial fibrillation.

^a^Participants with changes in BAPWV composition during follow-up were excluded (42,986).

#### Subgroup analysis

**Fig. 5:**
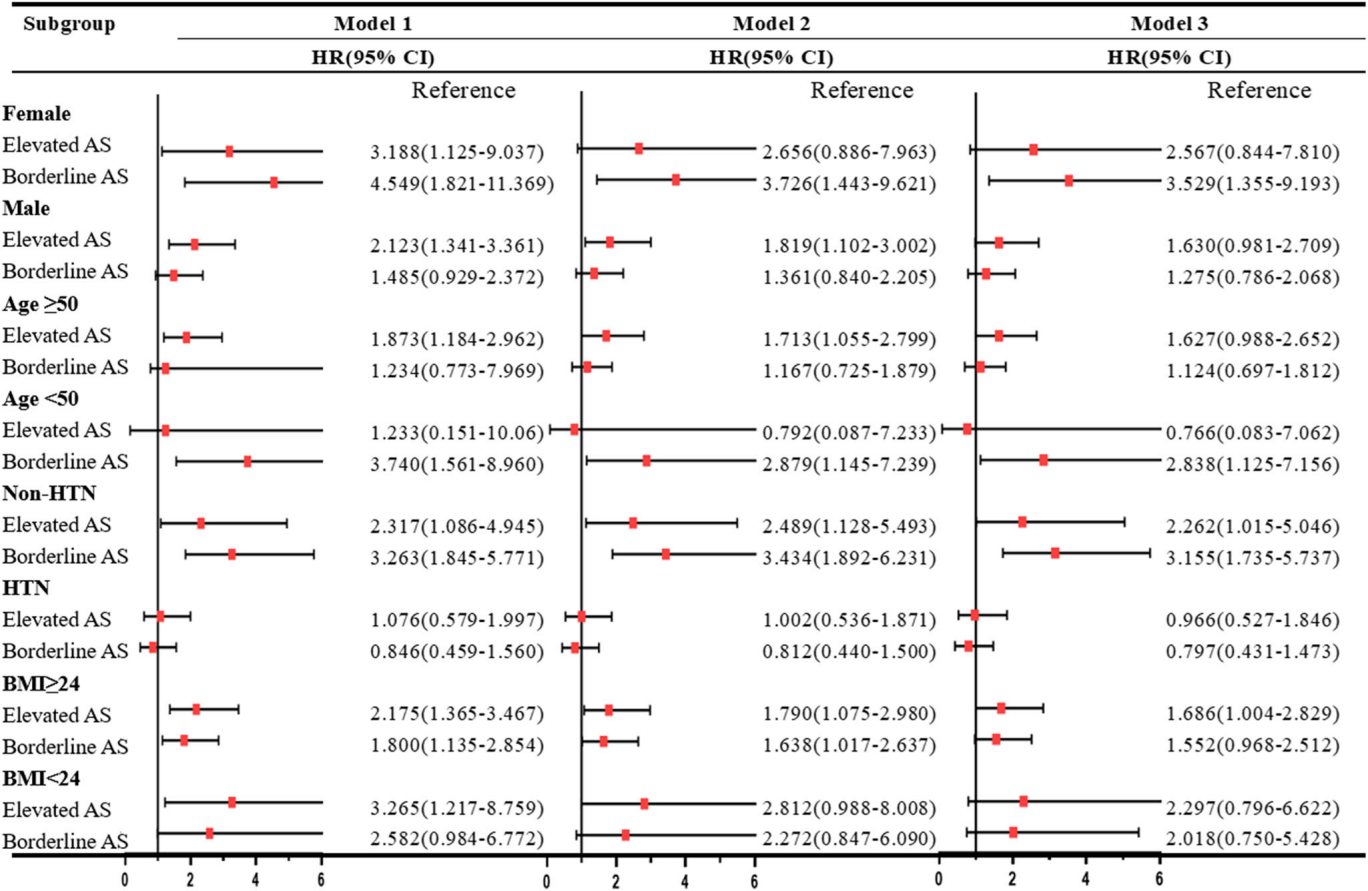
Association of arterial stiffness status combined with sex, age, BMI, and hypertension with AF. In each subgroup analysis, participants with normal brachial-ankle pulse were taken as reference. Model 1 was adjusted for age and sex at baseline; Model 2 was further adjusted for education, smoking, alcohol status, physical activity, salt intake, BMI, MAP, FBG, LDL-c, HDL-c and TC at baseline; Model 3 was further adjusted for antidiabetic, lipid-lowering and antihypertensive medications. AS arterial stiffness, BMI body mass index, HTN hypertension.

Subgroup analysis identified that amongst the patients in the elevated BMI group, the risk of AF in the elevated AS group was significantly higher than the reference group (HR for model 3: 1.69, 95% CI: 1.00–2.83; Fig. 5). For non-hypertensive patients, compared with the reference group, the risk of AF was also significantly increased in the borderline AS and elevated AS groups (HR for model 3: 3.16, 95 CI: 1.74–5.74; 2.26, 95% CI: 1.02–5.05, respectively). There were no significant differences in various subgroups e.g. age (50 years old as cut-off), gender, participants in non-elevated BMI group, and participants without hypertension.

## Discussion

Atrial fibrillation (AF) is one of the most common arrhythmias worldwide, which is associated with serious adverse events including thromboembolis. The management of AF is of utmost significance. Pulse wave velocity (PWV) represents the conduction velocity of pulse wave on arterial segments at a certain distance; it is an indirect expression of AS and reflects the compliance of large arteries. The higher the PWV, the worse the elasticity of vessels, and the higher the degree of AS^11^. The predictive value of PWV for AS and coronary artery disease (CAD) has been previously established, but its predictive value for new-onset AF remains unclear. In this study, we analyzed the relationship between baPWV and the risk of AF in consecutive patients who participated in the Kailuan cohort. Our principal findings are as follows: (1) the incidence of AF increased significantly in participants with higher baPWV; (2) for every 361 cm/s increase in baseline baPWV, the risk of AF increased by 21.7%; (3) there was a J-shaped association between baPWV and the risk of AF with an inflexion point at 1446 cm/s. These findings suggest that baPWV may be associated with new-onset AF.

Several studies have shown that there is a bidirectional relationship between AS and AF^12,13^, even forming a vicious circle between the two conditions. The mechanism underlying this link is complex and multifactorial, with several potential pathways implicated. Firstly, endothelial dysfunction, a hallmark of AS, is known to be strongly associated with AF^14,15^. Arterial stiffness is related to both endothelial dysfunction and inflammation^16^, which are key substrates for the occurrence and progression of AF^13^. Secondly, the role of inflammation in both AS and AF have been well described, with vascular stiffing as the potential trigger of an inflammatory process that eventually leads to the development of AF^17^. Thirdly, AS can also have a direct detrimental impact on left atrial geometry and function, which leads to AF^18^. Moreover, AS and its risk factors are associated with structural and electrical remodeling of the atrial, while atrial fibrosis is a consequence of the onset and progression of AF. Last but not least, the left ventricle hypertrophy and diastolic dysfunction associated with AS can favor the onset of AF^19^.

Compared to the traditional clinical indicators for the onset and progression of AF as reported in previous studies, baPWV has certain advantages. While cardiac structural and functional indicators such as left atrial diameter, left atrial volume index, and left ventricular ejection fraction assessed by echocardiography^20^ were also associated with the occurrence of AF, they were limited by equipment and operator expertise. In addition, numerous studies have found that C-reactive protein (CRP), interleukin-6 (IL-6), low-density lipoprotein (LDL), and other blood markers^21^ are also associated with the occurrence of AF, but they are limited by the invasiveness of venipunction. Therefore, it is necessary to develop an acceptable and simple protocol for AF prediction. Various studies have shown a strong correlation between AS and AF^12,22^. As a non-invasive examination, baPWV is clinically easy to measure, and it may be valuable in early identification and risk stratification of AF, given that it is a marker of AS and an independent risk factor for adverse cardiovascular events^23^. The present study discovered that baPWV is predictive of the occurrence of AF, representing a novel approach in the early identification of high-risk AF groups; thus, it holds great significance in improving AF outcomes at the population level.

Several methods exist for analyzing the structure and function of large arteries^24,25^, including echo-tracking systems, central pulse pressure analysis, and magnetic resonance that quantifies aortic distension and compliance^26^. However, the PWV measurement is currently considered the optimal measurement of AS because of its accuracy, simplicity, reproducibility. and predictive value. Although the use of carotid-femoral pulse wave velocity (cfPWV), a conventional method for evaluating AS, has been established for evaluating atherosclerotic diseases, its application in clinical settings is limited owing to technical difficulties. BaPWV is a simple and non-invasive alternative that requires only short measurement times, by only using cuffs on the limbs without the need for exposing the groin area or invasive examination. In addition, baPWV has been shown to correlate well with cfPWV^27^. The prognostic value of baPWV has been well validated in various clinical settings but not for new-onset AF previously^28^. In this study, the risk of new-onset AF increased with the increase of baPWV, which is consistent with the earlier conclusions that increased AS is a predisposing factor for AF, regardless it is reflected by aortic pulse wave velocity (aPWV)^29^ or estimated pulse wave velocity (ePWV)^30^. Compared with aPWV and ePWV, baPWV requires short measurement times and correlates well with AS in an invasive study^31^. These results indicate that baPWV is appropriate for use in routine examinations as well as for large clinical trials related to AF.

We also found a J-shaped association between baPWV and the risk of new-onset AF, which is consistent with a prior meta-analysis^22^. In this study, in the lower range of baPWV, the risk of AF remained low despite a steep gradient. However, when baPWV exceeded 1446 cm/s, the risk of AF increased with baPWV but with a relatively flattened gradient. 1446 cm/s was, therefore, the cut-off value for the risk of new-onset AF, and help identify high-risk patients. This underscoring the importance of maintaining optimal arterial health and reducing arterial stiffness to mitigate the risk of AF. This is aligned with the holistic and integrated approach to AF management, which encompasses beyond stroke prevention alone^32^. Such an approach is associated with improved clinical outcomes^33^ and is recommended in current guidelines^10,33,34^.

In this study, the association remained significant in the elevated BMI group but not in the non-elevated BMI group. The result suggested that weight control may be related to reduced risk of AS and AF, consistent with previous studies^35^. However, the results might also be due to the relatively insufficient power in the BMI < 24 group, with a relatively low incidence of AF in comparison (0.23% vs. 0.51%). The risk of AF was increased significantly in both the borderline AS group and the elevated AS group in the non-hypertensive population, as demonstrated in prior studies^26,30^. There was a linear relationship and consistency between ePWV and baPWV; notably, ePWV is significantly higher in non-hypertensive subjects than in the general population, also serving as a risk factor for AF^30^. Arterial stiffness may be a precursor rather than a consequence of hypertension^36^, indicating that AS plays a crucial role in the occurrence and development of AF.

Several limitations should be acknowledged. Firstly, all patients included in this study came from the Kailuan cohort study, with a large proportion of male (74.2%) participants. There might be selection bias and the results may not be generalizable to other population groups. Secondly, the diagnosis of AF during follow-up was based on physical examination findings or hospitalized ECGs, and some patients with paroxysmal atrial fibrillation might have been missed. Thirdly, echocardiographic data that are reported to be relevant to AF, such as left atrial size and left ventricular hypertrophy, were not available. Whilst baPWV showed promise in predicting AF, it is not clear whether baPWV adds beyond traditional factors in predicting AF, which requires further exploration. Moreover, the comparative analysis between baPWV and cfPWV, a conventional method for evaluating AS was not conducted since the cfPWV was not recorded. Finally, as with any long-term cohort study, there was inevitably a loss of follow-up and missing data.

In conclusion, in this large regional cohort study, baPWV was associated with the risk of new-onset AF, with participants in the elevated arterial stiffness group having a higher risk of AF.

## Data Availability

The data underlying this article were provided from the Kailuan cohort by permission. Data will be shared on request to the corresponding author(Shouling Wu) with permission of Kailuan cohort.
